# Targeted use of intraoperative frozen-section analysis lowers the frequency of completion thyroidectomy

**DOI:** 10.1093/bjsopen/zraa058

**Published:** 2021-04-01

**Authors:** J I Staubitz, I Elmrich, P B Musholt, R J A Cámara, F Watzka, H Dralle, C Sekulla, K Lorenz, T J Musholt, Prospective Evaluation Study Thyroid Surgery (PETS) 2 study group, C Vorländer, K Lorenz, C Blankenburg, C Geffcken, T Steinmüller, A Trupka, F Steinert, J Schabram, L Albrecht, C Marschall, C Orlitsch, K Holzner, J Feller, T Weber, D Kaltofen, D Simon, R Kube, K Schultz, M Sahm, J Obermeier, C Roth, K Janson, O Thomusch, H Meier, A Weinhold, N Müller, G Tonndorf, D Sinn, E Klein, G Henke, W Rampf, K Rendel, K Cupisti, K Holzer, D Grothe, L Axt, I Müller, W Probst, C Guhr, F Schischke, T Schwörig, M Konrad Hospital, J Fielitz, R Stets, M Liese, C- L Weiss, J Zaage, T Bräuer, J Weitz, A Huster, E Kidess, J Lautermann, N Kizilirmak, O Jannasch, H Bittscheidt, D Lehmann, K- P Kröll, T J Musholt, C Sonsnowska, Z Lorenc, B Dudesek, S Smutny, M Brauckhoff, E Bareck, R Köberle-Wührer

**Affiliations:** Section of Endocrine Surgery, Department of General, Visceral and Transplantation Surgery, University Medical Centre Mainz, Johannes Gutenberg University Mainz, Mainz, Germany; Section of Endocrine Surgery, Department of General, Visceral and Transplantation Surgery, University Medical Centre Mainz, Johannes Gutenberg University Mainz, Mainz, Germany; Section of Endocrine Surgery, Department of General, Visceral and Transplantation Surgery, University Medical Centre Mainz, Johannes Gutenberg University Mainz, Mainz, Germany; Institute for Medical Biometry, Epidemiology and Informatics, University Medical Centre Mainz, Johannes Gutenberg University Mainz, Mainz, Germany; Section of Endocrine Surgery, Department of General, Visceral and Transplantation Surgery, University Medical Centre Mainz, Johannes Gutenberg University Mainz, Mainz, Germany; Department of General, Visceral and Transplantation Surgery, University Medical Centre Essen, University Duisburg-Essen, Essen, Germany; Department of Visceral, Vascular and Endocrine Surgery, University Medical Centre Halle (Saale), Martin Luther University Halle-Wittenberg, Halle, Germany; Department of Visceral, Vascular and Endocrine Surgery, University Medical Centre Halle (Saale), Martin Luther University Halle-Wittenberg, Halle, Germany; Section of Endocrine Surgery, Department of General, Visceral and Transplantation Surgery, University Medical Centre Mainz, Johannes Gutenberg University Mainz, Mainz, Germany

## Abstract

**Background:**

The impact of intraoperative frozen section (iFS) analysis on the frequency of completion thyroidectomy for the management of thyroid carcinoma is controversial. Although specialized endocrine centres have published their respective results, there are insufficient data from primary and secondary healthcare levels. The aim of this study was to analyse the utility of iFS analysis.

**Methods:**

In the Prospective Evaluation Study Thyroid Surgery (PETS) 2 study, 22 011 operations for benign and malignant thyroid disease were registered prospectively in 68 European hospitals from 1 July 2010 to 31 December 2012. Group 1 consisted of 569 patients from University Medical Centre (UMC) Mainz, and group 2 comprised 21 442 patients from other PETS 2 participating hospitals. UMC Mainz exercised targeted but liberal use of iFS analysis for suspected malignant nodules. iFS analysis was compared with standard histological examination regarding the correct distinction between benign and malignant disease. The percentage of completion thyroidectomies was assessed for the participating hospitals.

**Results:**

iFS analysis was performed in 35.70 per cent of patients in group 1 *versus* 21.80 per cent of those in group 2 (risk ratio (RR) 1.6, 95 per cent c.i. 1.5 to 1.8; *P* < 0.001). Sensitivity of iFS analysis was 75.0 per cent in group 1 *versus* 63.50 per cent in group 2 (RR 1.2, 1.2 to 1.3; *P* = 0.040). Completion surgery was necessary in 8.10 per cent of patients in group 1 *versus* 20.8 per cent of those in group 2 (RR 0.4, 0.2 to 0.7; *P* = 0.001).

**Conclusion:**

iFS analysis is a useful tool in determining the appropriate surgical management of thyroid disease. Targeted use of iFS was associated with a significantly higher sensitivity for the detection of malignancy, and with a significantly reduced necessity for completion surgery.

## Teaser

Intraoperative frozen-section (iFS) analysis is discussed critically in the current literature, lacking a definite conclusion. The clinical value of iFS analysis for the surgical management of malignant thyroid disease is illustrated by this multicentre retrospective study .

## Resumen

**Antecedentes:**

El impacto de la biopsia intraoperatoria (*intraoperative frozen section*, iFS) sobre la frecuencia de completar la tiroidectomía en el tratamiento del carcinoma de tiroides es controvertido. Si bien los centros especializados en cirugía endocrina han publicado sus respectivos resultados, no hay datos suficientes para los niveles de atención primaria y secundaria. El objetivo de este estudio fue analizar la utilidad de la iFS.

**Métodos:**

En el estudio PETS 2 (*Prospective Evaluation Study Thyroid Surgery 2*), se registraron prospectivamente 22.011 operaciones por enfermedad tiroidea benigna y maligna en 68 hospitales europeos desde el 1 de julio de 2010 hasta el 31 de diciembre de 2012. El grupo 1 consistió en 569 pacientes de UMC Mainz; El grupo 2 comprendió 21.442 pacientes de otros hospitales participantes de PETS 2. En Mainz se realizó un uso selectivo, pero liberal, de la iFS para los nódulos malignos sospechosos. La iFS se comparó con la histología como patrón oro para distinguir correctamente entre enfermedad benigna y maligna. Se evaluó el porcentaje de tiroidectomías completadas por los hospitales participantes.

**Resultados:**

La iFS se realizó en el 35,7% en el grupo 1 *versus* 21,8% en el grupo 2 (riesgo relativo, RR 1,6; *P* < 0,001). La sensibilidad de la iFS fue del 75,0% en el grupo 1 *versus* 63,5% en el grupo 2 (RR 1,2; *P* = 0,040). Fue necesario completar la cirugía en el 8,0% del grupo 1 *versus* 20,8% del grupo 2 (RR 0,4; *P* < 0,001).

**Conclusión:**

La iFS es útil para determinar el tratamiento quirúrgico adecuado de la enfermedad tiroidea. El uso selectivo de la iFS se asoció con una sensibilidad significativamente mayor para detectar malignidad y con una necesidad significativamente menor de completar la cirugía.

## Introduction

Exclusion of malignancy is currently the predominant indication for thyroid surgery in Germany and other parts of Europe. The suspicion of thyroid malignancy is based mainly on growth rate, ultrasound patterns, elastography, results of ^99m^Tc scintigraphy (cold nodule)^1^ and, increasingly, on results of ^99m^Tc-sestamibi (MIBI) scintigraphy (mismatch)^2^. Multinodular goitre, compression symptoms and/or multifocal disease are frequently concomitant factors favouring thyroid resection. Negative experiences with misleading fine-needle aspiration cytology (FNAC) results, due to inexperience in performing the biopsy and in the cytopathological assessment, and to insufficient reimbursement for the FNAC procedure, are reasons why this diagnostic option is not used frequently in Germany and other parts of Europe, defying recommendations in guidelines^1^^,^^3^.

Intraoperative frozen-section (iFS) analysis is another option for the pathological evaluation of thyroid tumours, with the potential to influence the surgical strategy in the primary thyroid procedure, by switching to a more aggressive surgical approach in patients with malignancy. Despite ‘suspicion of malignancy’ being the predominant indication for thyroid surgery, and again in contrast to guidelines for the surgical management of thyroid disease^1^^,^^4^, at present iFS analysis is performed with a low frequency in Europe. Since the 1980s, the utility of iFS analysis has remained controversial^5–7^. Being hampered by the identification of capsular and vascular invasion, numerous authors^8–12^ have reported a low sensitivity for iFS analysis, in particular for the identification of follicular thyroid malignancy. Similar limitations, however, have also complicated preoperative FNAC, leading to ‘indeterminate’ diagnoses in 20–30 per cent of patients^13^.

The Prospective Evaluation Study Thyroid Surgery (PETS) 2, a prospective multicentre European study, was initiated to analyse the quality and pattern of medical care for thyroid disease. PETS 2 includes clinics from all levels of primary, secondary and tertiary care^14^, with the aim of assessing ‘service reality’ and establishing evidence for future recommendations and guidelines. The aim of the present analysis was to examine the sensitivity and potential impact of iFS analysis on the surgical management of thyroid malignancy in a ‘real world’ setting.

## Methods

PETS 2 included 68 European hospitals from Austria, Czech Republic, Germany, Norway and Poland, with the majority of centres being in Germany. A cross-section of low- and high-volume centres was included^14^. Consecutive patients undergoing a thyroid procedure from 1 July 2010 to 31 December 2012 were included in the study, with a follow-up period for complications until 31 December 2015. Perioperative and follow-up data were collected prospectively using a predefined questionnaire and transmitted in pseudonymized form.

To illustrate the differences between targeted but liberal *versus* infrequent use of iFS analysis, results from University Medical Centre (UMC) Mainz, Section of Endocrine Surgery (group 1) and those of the remaining hospitals participating in PETS 2 (group 2) were compared. In addition to the PETS 2 data, at UMC Mainz the initially intended resection strategy (unilateral *versus* bilateral lobe resection) as well as strategy changes were documented.

This work was carried out in accordance with the ethical standards of the Helsinki Declaration of 1975. Informed consent was obtained from all participants. The study was approved by the national ethical committee.

### Statistical analysis

Data were analysed with the IBM SPSS^®^ version 23 (IBM, Armonk, NY, USA). iFS analysis was compared with standard histological examination regarding the distinction between benign and malignant thyroid disease. Subgroup analysis, according to preoperative diagnosis, was also performed. Parameters of accuracy were calculated. The proportion of patients requiring completion surgery for intended radioiodine therapy^1^^,^^3^ (total thyroidectomy ± central lymphadenectomy in patients with incomplete, mostly unilateral, thyroid surgery) was analysed in both cohorts. Results were compared between the groups using Fisher’s exact test. Results with *P* < 0.050 were considered significant. Risk ratios (RRs) with 95 per cent c.i. are presented. Individual results for participating hospitals are presented in a funnel plot.

## Results

**Fig. 1:**
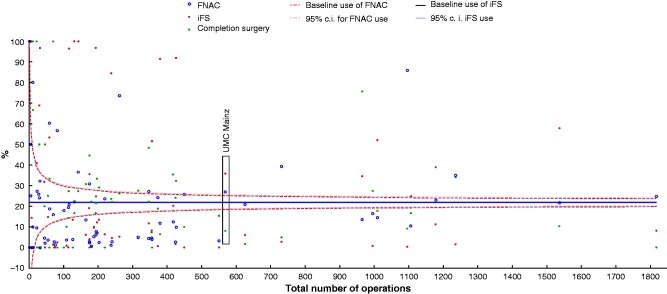
Funnel plot showing use of methods of analysis and resulting completion surgery rates for individual PETS 2 hospitals. Use of fine-needle aspiration cytology (FNAC) and intraoperative frozen-section (iFS) analysis, and completion surgery (percentage of patients with carcinoma), defined by the total number of operations in each Prospective Evaluation Study Thyroid Surgery (PETS) 2 hospital. UMC, University Medical Centre Mainz.

PETS 2 included 22 011 operations; 569 operations were performed at UMC Mainz (group 1) and 21 442 at the remaining hospitals (group 2) (*Table 1*). The interinstitutional comparison of the hospitals participating in PETS 2 found that both iFS analysis and FNAC were used more frequently than average at UMC Mainz (*Fig. 1*).

**Table 1:** Indications for surgical procedures performed in cohorts from University Medical Centre Mainz and the remaining PETS 2 hospitals

Preoperative diagnosis according to overall assessment*	Group 1 (UMC Mainz)		Group 2 (PETS 2)	
	Total (*n* = 569)	iFS analysis (*n* = 203)	Total (*n* = 21 442)	iFS analysis (*n* = 4681)
Benign lesion	465 (81.7)	128 (63.1)	19 387 (90.4, )	3636 (77.7)
Suspected malignancy	57 (10.0)	55 (27.1)	1203 (5.6, )	728 (15.6)
Malignancy^†^	38 (6.7)	20 (9.9)	401 (1.9)	223 (4.8)
Completion surgery for malignancy	9 (1.6)	0 (0)	451 (2.1)	94 (2.0)

Values in parentheses are percentages.

* Overall assessment includes clinical picture, sonography, elastography, scintigraphy, fine-needle aspiration cytology (with molecular genetic analyses), histology (for completion procedures only).

† Intraoperative frozen-section (iFS) analysis values include lymph node assessments. UMC, University Medical Centre; PETS, Prospective Evaluation Study Thyroid Surgery.

### Use of intraoperative frozen-section analysis and frequency of completion thyroidectomy

**Fig. 2:**
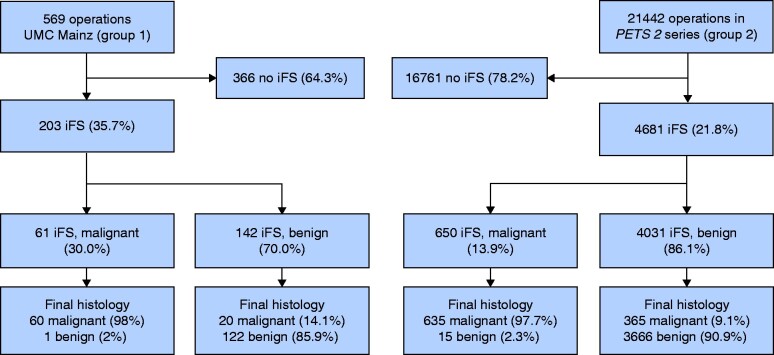
Patient flow diagram illustrating the use and results of intraoperative frozen-section analysis in relation to standard final histological examination UMC, University Medical Centre; PETS, Prospective Evaluation Study Thyroid Surgery; iFS, intraoperative frozen-section analysis.

**Fig. 3:**
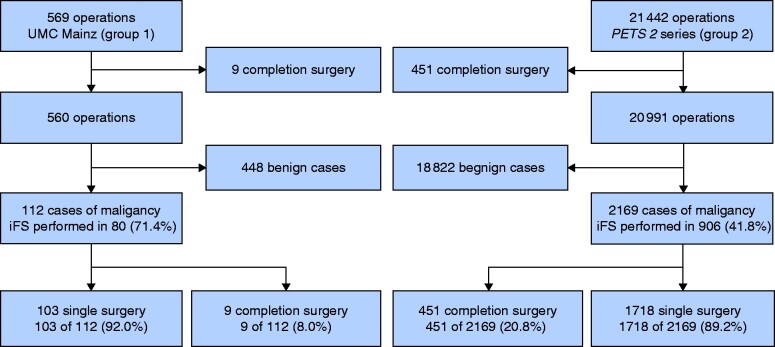
Patient flow diagram illustrating the proportion of patients receiving completion thyroidectomy after incomplete initial surgery in cancer cases UMC, University Medical Centre; PETS, Prospective Evaluation Study Thyroid Surgery; iFS, intraoperative frozen section.

iFS analysis was performed in 35.7 per cent of patients in group 1 and 21.8 per cent of those in group 2 (*Fig. 2*). In group 1, iFS analysis was performed in 27.5 per cent of patients considered before surgery to have benign disease, but which was deemed suspicious due to intraoperative findings (such as visual appearance, presence of desmoplasia, palpation), compared with 18.8 per cent in group 2. The proportion deemed before surgery to be ‘suspicious for malignancy’ was 10.0 per cent in group 1 and 5.6 per cent in group 2. For these patients, iFS analysis was performed in 27.1 per cent in group 1 *versus* 15.6 per cent in group 2 (*Table 1*). Of patients with malignant disease (later confirmed by histological examination), iFS analysis was conducted in 71.4 per cent in group 1, but in only 41.8 per cent in group 2 (*Fig. 3*).

**Fig. 4:**
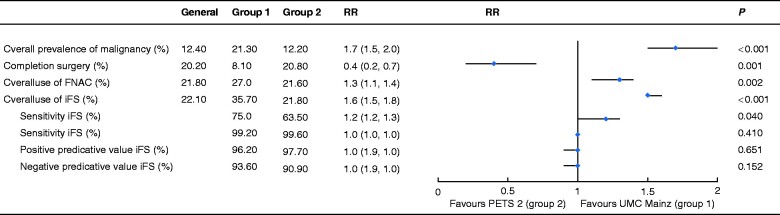
Forest plot comparing operative management characteristics at UMC Mainz (group 1) and the remaining PETS 2 hospitals (group 2) Values in parentheses are 95 per cent confidence intervals. RR, risk ratio; UMC, University Medical Centre; FNAC, fine-needle aspiration cytology; iFS, intraoperative frozen-section (analysis); PPV, positive predictive value; NPV, negative predictive value.

A significantly lower proportion of completion thyroidectomies was necessary in group 1 (9 of 112. 8.0 per cent) compared with group 2 (451 of 2169, 20.8 per cent) (RR 0.4, 95 per cent c.i. 0.2 to 0.7; *P* = 0.001) (*Fig. 4*), despite the significantly higher percentage of patients with thyroid carcinoma undergoing surgery at UMC Mainz (RR 1.7, 1.5 to 2.0; *P* < 0.001). Of note, 14.1 per cent (20 of 142) of the tumours judged as benign on iFS analysis at UMC Mainz were rediagnosed as malignant in the final histological assessment (*Fig. 2*). Seven of the nine cases of completion thyroidectomy in group 1 resulted from false-negative (incorrectly benign) iFS results. The underlying malignancies were four follicular thyroid carcinomas (FTCs) (including 1 oncocytic FTC and 1 minimally invasive FTC), two follicular variant papillary thyroid carcinomas (PTCs) and one ‘classical’ PTC.

### Predictive value of intraoperative frozen-section analysis

The sensitivity (correct detection of malignancy) of iFS analysis was significantly higher in group 1 than in group 2 (75.0 *versus* 63.5 per cent respectively) (RR 1.2, 95 per cent c.i. 1.2 to 1.3; *P* = 0.040). After adjusting for tumour prevalence, the positive predictive value (PPV) and negative predictive value of the two groups were similar (*Fig. 4* and *Table S1*).

In patients with thyroid disease diagnosed as benign before surgery, the sensitivity of iFS analysis in detecting malignancy was 65.2 per cent in group 1 and 40.5 per cent in group 2 (RR 1.6, *P* = 0.028; specificity 100 *versus* 99.6 per cent respectively) (data not shown).

### Extent of surgery

Bilateral thyroid lobe resection was the predominant resection strategy in both cohorts. In 8 per cent (6 of 74) of patients in group 1 with an intended unilateral procedure, the resection strategy was switched to bilateral lobe resection following iFS analysis, which correctly indicated malignancy (*Table 2*). In group 2, iFS analysis was performed in 18.8 per cent (3636 of 19 387) of patients who had disease classified as benign before surgery. Moreover, in 4 of 128 patients (3.1 per cent) in group 1 with thyroid disease diagnosed benign before surgery, central lymph node dissection was carried out, due to the diagnosis of malignancy following iFS analysis. In group 2, this occurred in only 26 of 3636 patients (0.7 per cent) (*Table 2*). In both groups, no false-positive iFS results preceded central lymph node resection. For this analysis, central lymphadenectomy was defined as resection of more than three lymph nodes from each side of the central lymph node compartment (compartments 1a and 1b^15^, level 6 (and 7) respectively^16^).

**Table 2:** Impact of intraoperative frozen-section analysis on surgical strategy

	Group 1 (UMC Mainz)		Group 2 (PETS 2)	
	**Total** **(*n* = 569)**	iFS analysis (*n* = 203)	Total (*n* = 21 442)	iFS (*n* = 4681)
**Surgical procedure intended**				
Unilateral surgery	155 (27.2)	74 (36.5)	n.a.	n.a.
Bilateral surgery	412 (72.4)	129 (63.5)	n.a.	n.a.
n.a.	2 (0.4)	0 (0)	21 442 (100)	4681 (100)
**Surgical procedure performed**				
Unilateral thyroid surgery	174 (30.6)	80 (39.4)	6492 (30.3)	1691 (36.1)
Bilateral thyroid surgery	394 (69.2)	123 (60.6)	14 561 (67.9)	2798 (59.8)
No thyroid resection	1 (0.2)	0 (0)	389 (1.8)	192 (4.1)
**Change of surgical management following results of iFS analysis**				
Change from unilateral to bilateral thyroid surgery	6 of 74 (8)		n.a.	
Change from intended thyroid surgery to thyroidectomy + central lymph node dissection^*^ in disease assumed benign before surgery^†^	4 of 128 (3.1)		26 of 3636 (0.7)	

Values in parentheses are percentages.

* Resection of more than three lymph nodes from each side of level 6^16^.

† Overall assessment includes clinical picture, sonography, elastography, scintigraphy, fine-needle aspiration cytology (with molecular genetic analyses), histology (for completion procedures only).

† Intraoperative frozen-section (iFS) analysis values include lymph node assessments. UMC, University Medical Centre; PETS, Prospective Evaluation Study Thyroid Surgery; n.a., not assessed.

### Histopathological findings

In both cohorts, most malignant tumours were PTC, as would be expected (*Table S2*). Malignant tumours that escaped detection in iFS analysis were PTC, including pT1a carcinoma and the follicular variant of PTC (*Table S2*). Among the malignant tumours escaping iFS detection, FTC was also common (20 per cent in group 1, 21.3 per cent in group 2). The relatively high proportion of medullary thyroid carcinoma among the undetected tumours was due to the fact that iFS analysis was used to determine the extent of disease.

### Subgroup analysis for fine-needle aspiration cytology categories

Preoperative FNAC analysis was available for 27.1 per cent of patients in group 1 and 21.6 per cent of those in group 2 (*Table S3*). The contemporaneous availability of iFS and FNAC results allowed for an analysis of iFS sensitivity for different Bethesda categories (*Fig. S1* and *Table S4*). The category associated with the highest sensitivity for iFS analysis was Bethesda category IV: 82.3 per cent for group 1 and 83.3 per cent for group 2 (*Table S4*).

## Discussion

The sensitivity of iFS analysis was significantly higher in the UMC Mainz group (75.0 per cent *versus* 63.5 per cent in the PETS 2 cohort), and completion surgery was performed significantly more often in the PETS 2 cohort (group 2) (20.8 per cent *versus* 8.1 per cent in the UMC Mainz group). One reason for the significantly lower risk of completion surgery at UMC Mainz was the more frequent use of iFS analysis (35.7 per cent *versus* 21.8 per cent in group 2), particularly the intentional use of iFS analysis for thyroid nodules assumed before surgery to be benign, but deemed suspicious in the intraoperative setting. The decision to perform iFS of a respective nodule was influenced by the clinical picture (such as patient age, node size, growth rate), sonographic patterns (preoperative ultrasound imaging by the operating surgeon), palpatory findings (hardness, elasticity, calcifications) and—especially visible in nodules located near the thyroid capsule—the presence of desmoplastic alterations (such as dense fibrosis around the nodule, star-shaped fibrosis). Cysts or nodules featuring visible colloid areas were judged less suspicious and did not undergo iFS analysis. Enlarged or otherwise suspicious lymph nodes were assessed routinely by means of iFS analysis.

The targeted selection of nodules subjected to iFS analysis is illustrated by the result that in group 1 iFS was conducted in 71.4 per cent of patients with confirmed malignancy at final histology, compared with 41.9 per cent in the remaining PETS 2 cohort. Selecting nodules or lymph nodes for iFS analysis is dependent on the experience of the surgeon—experience that comes from having been exposed frequently to iFS results during training and professional career.

The RR of sensitivity significantly favoured the UMC Mainz cohort, whereas the specificity of iFS analysis was similar for the two groups, and a direct influence of carcinoma prevalence on iFS sensitivity and specificity was excluded statistically. Routine use of iFS develops and maintains the expertise of the pathologists involved, allowing for a higher sensitivity. Although rare, false-negative iFS results were documented at UMC Mainz, leading to seven completion thyroidectomies. Addressing this problem, from 2013 onward, UMC Mainz performed fast paraffin embedding (12–36 h) in patients with ‘suspicious’ iFS results (stromal desmoplasia, homogeneous follicular architecture and hypercellularity), allowing for the performance of completion thyroidectomy within 72 h of primary surgery; within the period associated with less morbidity and an improved oncological outcome^17^.

In 2015, Hosseini and colleagues^18^ reported a 73 per cent reduction of secondary surgery in patients with follicular lesions on preoperative FNAC, influenced by the results of iFS analysis. Estebe and co-workers^19^ found that, independent of Bethesda categories, iFS analysis contributed to a reduction of secondary surgery. In addition, in PTC diagnosed before surgery , Hong *et al*.^20^ and Park and Lee^21^ reported the value of iFS analysis in determining the extent of extracapsular invasion, which potentially influences the resection strategy.

The literature suggests that the sensitivity of iFS depends essentially on the histological subtype. Whereas 21 per cent of follicular lesions were detected, non-follicular lesions (primarily PTC) were detected with a sensitivity of 66 per cent in a meta-analysis by Peng and Wang^22^. From this meta-analysis of the literature from 1982 to 2007, the authors concluded that the sensitivity of iFS analysis to detect FTC was significantly lower than the sensitivity provided by preoperative FNAC (21 *versus* 69 per cent respectively). Yet, for this analysis, the diagnosis of ‘follicular neoplasm’ by FNAC was considered test-positive, whereas for a test-positive result in iFS analysis precise criteria of malignancy (capsular and vascular invasion) had to be fulfilled. Consequently, specificity and PPV significantly favoured iFS diagnosis (specificity 99 per cent *versus* 60 per cent for FNAC; PPV 86 *versus* 35 per cent respectively)^22^. In 21 studies that did not differentiate between follicular and non-follicular lesions, iFS analysis appeared significantly superior to FNAC diagnosis for the above-mentioned measures of accuracy^22^. The detected sensitivity (71 ± 13 per cent) was similar to the results of the present analysis. The present study illustrates the diagnostic restriction of iFS analysis for the correct evaluation of the follicular variant of PTC, papillary microcarcinoma and FTC. Cohen and co-workers^23^ also reported particular difficulties for assessment of the follicular variant of PTC.

In addition to differences in the frequency of iFS analysis, the significantly higher number of preoperative FNAC examinations performed in the cohort undergoing surgery at UMC Mainz contributed to the reduction in completion thyroidectomy rates. Cetin *et al.*^24^ reported the utility of iFS analysis (sensitivity 72.9 per cent, specificity 100 per cent) in thyroid nodules with a FNAC diagnosis of ‘suspicious for malignant disease’. Similarly, Roychoudhury and colleagues^25^ and Cohen *et al.*^23^ reported the utility of iFS analysis in nodules of Bethesda category V, which, in contrast, was not observed by Huang and co-workers^26^. In the present study, a sensitivity for iFS analysis of 82.3 per cent for nodules of Bethesda category V was documented at UMC Mainz, and 83.3 per cent in the PETS 2 cohort. Posillico *et al.*^27^ reported iFS analysis to be an important tool for determining the extent of thyroid surgery in patients with nodular thyroid and preoperative FNAC results categorized as atypia/follicular lesion of undetermined significance (Bethesda category III).

The increasing use of molecular testing of FNAC samples will further refine preoperative diagnosis in the future, directing iFS analysis to become an additional complementary tool for acquiring information on tumour size, focality, lymph node affection or extracapsular growth^28^^,^^29^.

To optimize the preoperative and intraoperative diagnosis of differentiated thyroid carcinoma, multimodal assessment including sonography, elastography, scintigraphy, FNAC with molecular genetic analysis, and iFS analysis is of crucial importance. The position of iFS analysis within the framework of this multimodal assessment is a central one, complemented by the experience of the thyroid surgeon in evaluating the examinations performed before surgery, and especially during surgery.

## Collaborators

Members of the PETS 2 study group:

Germany: C. Vorländer (Department of Endocrine Surgery, Bürgerhospital Frankfurt am Main, Frankfurt am Main); K. Lorenz (Department of General, Visceral and Vascular Surgery, University Hospital Halle/Saale, Halle/Saale),; C. Blankenburg (Department of Surgery, Hospital St Josef-Stift, Dresden); C. Geffcken (Department of Surgery, Henriettenstiftung, Hannover); T. Steinmüller (Department of General Surgery, DRK Hospitals Berlin Westend, Berlin); A. Trupka (Department of Surgery, Hospital Starnberg, Starnberg); F. Steinert (Department of General and Visceral Surgery, Helios Hospital Schkeuditz, Schkeuditz); J. Schabram (Department of General and Visceral Surgery, St Josefs Hospital Giessen, Giessen); L. Albrecht (Department of Surgery, Hospital St Marienstift Magdeburg, Magdeburg); C. Marschall (Department of Thyroid Surgery, Eichsfeld Hospital, Reifenstein); C. Orlitsch (Department of Surgery, Maria Theresia Hospital, Munich); K. Holzner (Department of Surgery, Vivantes Wenckebach Hospital, Berlin); J. Feller (Department of General and Visceral Surgery, Sana Hospital Lichtenberg, Berlin-Lichtenberg); T. Weber (Department of General, Visceral and Transplantation Surgery, University Hospital of Ulm, Ulm); D. Kaltofen (Department of General and Visceral Surgery, Hospital Chemnitz, Chemnitz); D. Simon (Department of General and Visceral Surgery, Evangelisches Bethesda Johanniter Hospital Duisburg, Duisburg); R. Kube (Department of Surgery, Carl Thiem Hospital Cottbus, Cottbus); K. Schultz (Department of Surgery, St Hedwig Hospital Berlin, Berlin); M. Sahm (Department of Surgery, DRK Hospital Berlin Köpenick, Berlin); J. Obermeier (Department of Surgery, Hospital Dortmund, Dortmund); C. Roth (Department of General and Visceral Surgery, Hospital Kulmbach, Kulmbach); K. Janson (Department of General and Visceral and Thoracic Surgery, Hospital Sozialstiftung Bamberg, Bamberg); O. Thomusch (Department of General and Visceral Surgery, University Hospital of Freiburg, Freiburg); H. Meier (Department of Surgery, Sana Hospital Benrath, Düsseldorf); A. Weinhold (Department of Surgery, Diakonie Hospital Halle, Halle/Saale); N. Müller (Department of Visceral Surgery, Westpfalz Hospital, Kaiserslautern); G. Tonndorf (Department of General and Visceral Surgery, Asklepios Hospital Altona, Hamburg); D. Sinn (Section of Surgery St Martinus Hospital Olpe, Katholische Hospitalgesellschaft Südwestfalen, Olpe); E. Klein (Department of General and Visceral Surgery, Hospital Lüdenscheid, Lüdenscheid); G. Henke (Department of General and Visceral Surgery, Städtisches Hospital Dresden Friedrichstadt, Dresden); W. Rampf (Department of Surgery, Hospital 14 Nothelfer, Weingarten); K. Rendel (Department of General and Visceral Surgery, Hospital Magdeburg, Magdeburg); K. Cupisti (Department of General and Visceral Surgery, University Hospital of Düsseldorf, Düsseldorf); K. Holzer (Department of General and Visceral Surgery, University Hospital of Universitätsklinikum Frankfurt am Main, Frankfurt am Main); D. Grothe (Department of General and Visceral Surgery, Christliches Hospital Melle, Melle); L. Axt (Department of General, Visceral and Transplantation Surgery, Hospital Augsburg, Augsburg); I. Müller (Department of General and Visceral Surgery at Mittweida, Landkreis Mittweida Hospital, Mittweida); W. Probst (Department of General and Visceral Surgery, Ammerland Hospital, Westerstede); C. Guhr (Department of Surgery, Hospital Schönebeck, Schönebeck); F. Schischke (Department of General and Visceral Surgery, DRK Hospital Luckenwalde, Luckenwalde); T. Schwörig (Klinik für Allgemein und Visceralchirurgie, Klinikum Niederlausitz, Senftenberg); M. Konrad Hospital (Department of General and Visceral Surgery, Neustadt Betriebs KG, Neustadt in Holstein); J. Fielitz (Department of Surgery, Paracelsus Hospital Reichenbach, Reichenbach); R. Stets (Department of General, Visceral and Vascular Surgery, Hospital Martha-Maria Halle-Dölau, Halle/Saale); M. Liese (Department of Surgery, Oder Spree Hospital, Beeskow); C.-L. Weiss (Department of General and Visceral Surgery, Hospital St Elisabeth and St Barbara, Halle/Saale); J. Zaage (Department of General Surgery, Hospital Bergmannstrost, Halle/Saale); T. Bräuer (Department of General and Visceral Surgery, Hospital Mittleres Erzgebirge Zschopau, Zschopau); J. Weitz (Department of Visceral, Thoracic and Vascular Surgery, University Hospital Carl Gustav Carus, Dresden); A. Huster (Department of Surgery, Hospital Freiberg, Freiberg); E. Kidess (Department of General, Thoracic, Vascular and Transplantation Surgery, University Hospital of Rostock, Rostock); J. Lautermann (Department of Ear Nose Throat Surgery, Hospital Martha-Maria Halle-Dölau, Halle/Saale); N. Kizilirmak (Department of General and Visceral Surgery, Marien Hospital Witten, Witten); O. Jannasch (Department of General, Visceral and Vascular Surgery, University Hospital of Otto von Guericke University, Magdeburg); H. Bittscheidt (Department of General and Visceral Surgery, Sana Hospital Hameln-Pyrmont, Hameln); D. Lehmann (Department of General and Visceral Surgery, Suedharz Hospital Nordhausen, Nordhausen); K.-P. Kröll (Department of General and Visceral Surgery, Hochwaldkrankenhaus, Bad Nauheim); T. J. Musholt (Section of Endocrine Surgery, Department of General, Visceral and Transplantation Surgery, UMC Mainz, ,Mainz).

Poland: C. Sonsnowska (Oddział Chirurgii Ogólnej, Szpital Wojewódzki w Poznaniu, Poznań); Z. Lorenc (Specjalistyczny Nr 5 im. św. Barbary, Wojewódzki Szpital, Sosnowiec).

Czech Republik: B. Dudesek (Tomáš Baťa University, Zlín); S. Smutny (Department of Surgery, Oblastni Nemocnice Pribram, Pribram).

Norway: M. Brauckhoff (Department of Surgery, University of Bergen Haukeland University Hospital, Bergen).

Austria: E. Bareck (Department of Surgery, Hospital Vienna Neustadt, Vienna Neustadt); R. Köberle-Wührer (Department of General and Thoracic Surgery, Landeskrankenhaus Feldkirch, Feldkirch).

## Supplementary Material

zraa058_Supplementary_Data

## Notes

Presented to the European Society of Endocrine Surgeons, Amsterdam, the Netherlands, May 2018

## References

[1] Dralle H, Musholt TJ, Schabram J, Steinmuller T, Frilling A, Simon D et al German Association of Endocrine Surgeons practice guideline for the surgical management of malignant thyroid tumors. Langenbecks Arch Surg 2013;398:347–375 23456424 10.1007/s00423-013-1057-6

[2] Treglia G, Caldarella C, Saggiorato E, Ceriani L, Orlandi F, Salvatori M et al Diagnostic performance of ^99m^Tc-MIBI scan in predicting the malignancy of thyroid nodules: a meta-analysis. Endocrine 2013;44:70–78 23529672 10.1007/s12020-013-9932-z

[3] Haugen BR, Alexander EK, Bible KC, Doherty GM, Mandel SJ, Nikiforov YE et al 2015 American Thyroid Association management guidelines for adult patients with thyroid nodules and differentiated thyroid cancer: the American Thyroid Association Guidelines Task Force on thyroid nodules and differentiated thyroid cancer. Thyroid 2016;26:1–133 26462967 10.1089/thy.2015.0020 PMC4739132

[4] Musholt TJ, Bockisch A, Clerici T, Dotzenrath C, Dralle H, Goretzki PE et al; Leitliniengruppe der CAEK. Update of the S2k guidelines : surgical treatment of benign thyroid diseases. Chirurg 2018;89:699–709 29876616 10.1007/s00104-018-0653-y

[5] Keller MP, Crabbe MM, Norwood SH. Accuracy and significance of fine-needle aspiration and frozen section in determining the extent of thyroid resection. Surgery 1987;101:632–635 3576454

[6] Hamburger JI, Hamburger SW. Declining role of frozen section in surgical planning for thyroid nodules. Surgery 1985;98:307–312 4023923

[7] Layfield LJ, Mohrmann RL, Kopald KH, Giuliano AE. Use of aspiration cytology and frozen section examination for management of benign and malignant thyroid nodules. Cancer 1991;68:130–134 2049733 10.1002/1097-0142(19910701)68:1<130::aid-cncr2820680124>3.0.co;2-9

[8] Alonso N, Lucas A, Salinas I, Castella E, Sanmarti A. Frozen section in a cytological diagnosis of thyroid follicular neoplasm. Laryngoscope 2003;113:563–566 12616215 10.1097/00005537-200303000-00031

[9] Bollig CA, Lesko D, Gilley D, Dooley LM. The futility of intraoperative frozen section in the evaluation of follicular thyroid lesions. Laryngoscope 2018;128:1501–1505 28990674 10.1002/lary.26937

[10] LiVolsi VA, Baloch ZW. Use and abuse of frozen section in the diagnosis of follicular thyroid lesions. Endocr Pathol 2005;16:285–293. 16627916 10.1385/ep:16:4:285

[11] Carling T, Udelsman R. Follicular neoplasms of the thyroid: what to recommend. Thyroid 2005;15:583–587 16029125 10.1089/thy.2005.15.583

[12] Kennedy JM, Robinson RA. Thyroid frozen sections in patients with preoperative FNAs: review of surgeons' preoperative rationale, intraoperative decisions, and final outcome. Am J Clin Pathol 2016;145:660–665 27124950 10.1093/ajcp/aqw042

[13] Bongiovanni M, Spitale A, Faquin WC, Mazzucchelli L, Baloch ZW. The Bethesda system for reporting thyroid cytopathology: a meta-analysis. Acta Cytol 2012;56:333–339 22846422 10.1159/000339959

[14] Thomusch O, Sekulla C, Billmann F, Seifert G, Dralle H, Lorenz K. Risk profile analysis and complications after surgery for autoimmune thyroid disease. Br J Surg 2018;105:677–685 29579336 10.1002/bjs.10770

[15] Dralle H, Damm I, Scheumann GF, Kotzerke J, Kupsch E, Geerlings H et al Compartment-oriented microdissection of regional lymph nodes in medullary thyroid carcinoma. Surg Today 1994;24:112–121 8054788 10.1007/BF02473391

[16] Robbins KT, Shaha AR, Medina JE, Califano JA, Wolf GT, Ferlito A et al Consensus statement on the classification and terminology of neck dissection. Arch Otolaryngol Head Neck Surg 2008;134:536–538 18490577 10.1001/archotol.134.5.536

[17] Glockzin G, Hornung M, Kienle K, Thelen K, Boin M, Schreyer AG et al Completion thyroidectomy: effect of timing on clinical complications and oncologic outcome in patients with differentiated thyroid cancer. World J Surg 2012;36:1168–1173 22366982 10.1007/s00268-012-1484-5

[18] Hosseini M, Alizadeh Otaghvar HR, Tizmaghz A, Shabestanipour G, Arvaneh S. Evaluating the accuracy of fine needle aspiration and frozen section based on permanent histology in patients with follicular lesions. Med J Islam Repub Iran 2015;29:239 26793630 PMC4715402

[19] Estebe S, Montenat C, Tremoureux A, Rousseau C, Bouilloud F, Jegoux F. Limitation of intraoperative frozen section during thyroid surgery. Eur Arch Otorhinolaryngol 2017;274:1671–1676 27913858 10.1007/s00405-016-4398-2

[20] Hong JC, Seo JW, Jang AL, Suh SH, Pak MG, Han SH et al The utility of intra-operative frozen section for the evaluation of microscopic extrathyroidal extension in papillary thyroid carcinoma. Clin Otolaryngol 2017;42:1167–1171 28166397 10.1111/coa.12843

[21] Park YM, Lee BJ. Intraoperative surgical decision using intraoperative frozen section in papillary thyroid cancer: reply. World J Surg 2015;39:1856–1857 25651966 10.1007/s00268-015-2991-y

[22] Peng Y, Wang HH. A meta-analysis of comparing fine-needle aspiration and frozen section for evaluating thyroid nodules. Diagn Cytopathol 2008;36:916–920 18855886 10.1002/dc.20943

[23] Cohen MA, Patel KR, Gromis J, Kutler DI, Kuhel WI, Stater BJ et al Retrospective evaluation of frozen section use for thyroid nodules with a prior fine needle aspiration diagnosis of Bethesda II–VI: the Weill Cornell Medical College experience. World J Otorhinolaryngol Head Neck Surg 2015;1:5–10 29204534 10.1016/j.wjorl.2015.09.002 PMC5698504

[24] Cetin B, Aslan S, Hatiboglu C, Babacan B, Onder A, Celik A et al Frozen section in thyroid surgery: is it a necessity? Can J Surg 2004;47:29–33 14997922 PMC3211814

[25] Roychoudhury S, Souza F, Gimenez C, Glass R, Cocker R, Chau K et al Utility of intraoperative frozen sections for thyroid nodules with prior fine needle aspiration cytology diagnosis. Diagn Cytopathol 2017;45:789–794 28603866 10.1002/dc.23765

[26] Huang J, Luo J, Chen J, Sun Y, Zhang C, Xu K et al Intraoperative frozen section can be reduced in thyroid nodules classified as Bethesda categories V and VI. Sci Rep 2017;7:5244 28701706 10.1038/s41598-017-05459-x PMC5507921

[27] Posillico SE, Wilhelm SM, McHenry CR. The utility of frozen section examination for determining the extent of thyroidectomy in patients with a thyroid nodule and ‘atypia/follicular lesion of undetermined significance’. Am J Surg 2015;209:552–556 25554703 10.1016/j.amjsurg.2014.09.026

[28] Krane JF, Cibas ES, Alexander EK, Paschke R, Eszlinger M. Molecular analysis of residual ThinPrep material from thyroid FNAs increases diagnostic sensitivity. Cancer Cytopathol 2015;123:356–361 25926393 10.1002/cncy.21546

[29] Nikiforov YE, Ohori NP, Hodak SP, Carty SE, LeBeau SO, Ferris RL et al Impact of mutational testing on the diagnosis and management of patients with cytologically indeterminate thyroid nodules: a prospective analysis of 1056 FNA samples. J Clin Endocrinol Metab 2011;96:3390–3397 21880806 10.1210/jc.2011-1469 PMC3205883

