# Supplementary material for: Targeted use of intraoperative frozen-section analysis lowers the frequency of completion thyroidectomy
Source: BJS Open. 2021 Apr 1;5(2):zraa058. doi: 10.1093/bjsopen/zraa058 (PMC8045471; doi:10.1093/bjsopen/zraa058)
Supplement: zraa058_Supplementary_Data [file zraa058_supplementary_data.zip › Supplementary Tables.docx]

**Supplementary Tables**

**Supplementary Table 1 Measures of accuracy of intraoperative frozen section analysis (results in %, 95%-confidence intervals)**

|  | **Group 1: UMC Mainz ^a^** | **Group 2: PETS 2 ^b^** | **Risk ratio** | **p-value** |
| --- | --- | --- | --- | --- |
| Sensitivity | 75.0 (64.1-84.0) | 63.5 (60.4-66.5) | 1.2 (1.0-1.3) | 0.040* |
| Specificity | 99.2 (95.5-10.0) | 99.6 (99.3-99.8) | 1.0 (1.0-1.0) | 0.410 |
| Positive predictive value | 98.4 (91.2-100) | 97.7 (96.2-98.7) | 1.0 (1.0-1.0) | 1.000 |
| Negative predictive value | 85.9 (79.1-91.2) | 90.9 (90.0-91.8) | 0.9 (0.9-1.0) | 0.053 |
| Prevalence of malignancy | 39.4 (32.6-46.5) | 21.4 (20.2-22.6) | 1.8 (1.5-2.2) | <0.001* |
| Positive predictive value with adjusted prevalence ^c^ | 96.2 (88.1-99.1) | 97.7 (96.2-98.7) | 1.0 (0.9-1.0) | 0.651 |
| Negative predictive value with adjusted prevalence ^c^ | 93.6 (88.4-97.2) | 90.9 (90.0-91.8) | 1.0 (0.9-1.0) | 0.152 |

^a^ University Medical Center Mainz

^b^ Prospective Evaluation Study Thyroid Surgery 2

^c^ predictive values adjusted according to the prevalence of thyroid cancer in group 2

**Supplementary Table 2 Malignant thyroid disease in the cohorts from the UMC Mainz and the remaining PETS 2 hospitals (results in %, N)**

|  | **Group 1: UMC Mainz** ^a^ | | **Group 2: PETS 2 series** ^b^ | |
| --- | --- | --- | --- | --- |
|  | total | iFS | total | iFS |
| Women | 69.8, 397/569 | 70.9, 144/203 | 74.8, 16034/21442 | 70.3, 3291/4681 |
| Previous thyroid surgery | 12.7, 72/569 | 10.8, 22/203 | 9.6, 2064/21442 | 11.4, 535/4681 |
| - for benign disease | 54.2, 39/72 | 45.5, 10/22 | 55.4, 1144/2064 | 49.5, 265/535 |
| - for malignant disease | 34.7, 25/72 | 40.9, 9/22 | 32.5, 671/2064 | 44.3, 237/535 |
| - for unknown disease | 11.1, 8/72 | 13.6, 3/22 | 12.1, 249/2064 | 6.2, 33/535 |
| patients with the diagnosis of malignant tumors in histology (gold standard) = prevalence | 21.3, 121/569 | 39.4, 80/203 | 12.2, 2620/21442 | 21.4, 1000/4681 |
| Tumor entities detected by histology (gold standard) (N) | 121 | 80 | 2715 ^c^ | 1073 ^c^ |
| - Papillary thyroid carcinoma | 57.8, 70/121 | 52.5, 42/80 | 70.3, 1909/2715 | 61.9, 664/1073 |
| - - Papillary thyroid microcarcinoma, pT1a | 14.9, 18/121 | 10.0, 8/80 | 21.0, 570/2715 | 15.7, 168/1073 |
| - Medullary thyroid carcinoma | 21.5, 26/121 | 31.2, 25/80 | 11.2, 305/2715 | 16.2, 174/1073 |
| - Follicular thyroid carcinoma | 13.2, 6/121 | 11.3, 9/80 | 13.4, 363/2715 | 15.3, 164/1073 |
| - - Minimally invasive follicular thyroid carcinoma | 3.3, 4/121 | 5.0, 4/80 | 4.2, 114/2715 | 3.8, 41/1073 |
| - - Widely invasive follicular thyroid carcinoma | 0.0, 0/121 | 0.0, 0/80 | 1.8, 49/2715 | 2.3, 25/1073 |
| - Poorly differentiated thyroid carcinoma | 3.3, 4/121 | 5.0, 4/80 | 0.8, 23/2715 | 0.9, 10/1073 |
| - Anaplastic thyroid carcinoma | 0.0, 0/121 | 0.0, 0/80 | 1.5, 41/2715 | 2.6, 28/1073 |
| - Lymphoma | 0.8, 1/121 | 0.0, 0/80 | 0.7, 18/2715 | 0.7, 7/1073 |
| - Metastases to the thyroid | 1.7, 2/121 | 0.0, 0/80 | 1.5, 41/2715 | 1.7, 19/1073 |
| - Other malignant entities | 1.7, 2/121 | 0.0, 0/80 | 0.6, 15/2715 | 0.7, 7/1073 |
| Patients with malignant tumors, undetected by intraoperative frozen section | 9.9, 20/203 | | 7.8, 365/4681 | |
| Tumor entities undetected by intraoperative frozen section (N) | 20 | | 394 § | |
| - Papillary thyroid carcinoma | 45.0, 9/20 | | 66.5%, 262/394 | |
| - - Papillary thyroid microcarcinoma, pT1a | 10.0, 2/20 | | 28.2, 111/394 | |
| - - Papillary thyroid carcinoma, follicular variant | 15.0, 3/20 | | not assessed | |
| - Follicular thyroid carcinoma | 20.0, 4/20 | | 21.3, 84/394 | |
| - - Minimally invasive follicular thyroid carcinoma | 10.0, 2/20 | | 6.9, 27/394 | |
| - - Widely invasive follicular thyroid carcinoma | 0.0, 0/20 | | 2.8, 11/394 | |
| - Medullary thyroid carcinoma | 30.0, 6/20 | | 10.4, 41/394 | |
| - Poorly differentiated thyroid carcinoma | 5.0, 1/20 | | 0.2, 1/394 | |
| - Lymphoma | 0.0, 0/20 | | 0.8, 3/394 | |
| - Other entities | 0.0, 0/20 | | 0.8, 3/394 | |

^a^ University Medical Center Mainz

^b^ Prospective Evaluation Study Thyroid Surgery 2

^c^ simultaneous existence of different malignant tumors in the same patient

**Supplementary Table 3 Indications for surgical procedures performed in cohorts from the UMC Mainz and the remaining PETS 2 hospitals (results in %, N)**

|  | **Group 1: UMC Mainz** ^a^ | | **Group 2: PETS 2** ^b^ | |
| --- | --- | --- | --- | --- |
|  | **total**  **(n=569)** | **cases with iFS**  **(n=203)** | **total**  **(n=21442)** | **cases with iFS (n=4681)** |
| Results fine needle aspiration cytology | | | | |
| - Total | 27.1, 154 | 39.4, 80 | 21.6, 4635 | 22.9, 1070 |
| - Non-diagnostic (Bethesda category I) | 5.2, 8/154 | 5.0, 4/80 | 4.1, 191/4635 | 6.4, 69/1070 |
| - Benign (Bethesda category II) | 55.8, 86/154 | 42.5, 34/80 | 68.4, 3169/4635 | 45.6, 488/1070 |
| - Follicular neoplasm (Bethesda category IV) | 16.9, 26/154 | 20.0, 16/80 | 14.1, 654/4635 | 21.6, 231/1070 |
| - Suspicious for malignancy (Bethesda category V) | 22.1, 34/154 | 32.5, 26/80 | 13.4, 621/4635 | 26.4, 282/1070 |

^a^ University Medical Center Mainz

^b^ Prospective Evaluation Study Thyroid Surgery 2

**Supplementary Table 4 Measures of accuracy of index test intraoperative frozen section for subgroups classified by results of preoperative overall assessment (results in %, 95%-confidence intervals)**

|  | **Group 1: UMC Mainz** ^a^ | **Group 2: PETS 2** ^b^ | **Risk ratio** | **p-value** |
| --- | --- | --- | --- | --- |
| Diagnosis according to FNAC ^c^: benign (Bethesda category II) | | | | |
| - Sensitivity | 62.5 (31.0-86.3) | 42.2 (30.9-54.4) | 1.5 (0.8-2.7) | 0.453 |
| - Specificity | 100 (87.1-100) | 99.8 (98.7-99.9) | 1.0 (1.0-1.0) | 1.000 |
| Diagnosis according to FNAC: follicular neoplasm (Bethesda category III) | | | | |
| - Sensitivity | 80.0 (26.3-100) | 66.7 (42.6-88.6) | 1.2 (0.8-1.7) | 0.486 |
| - Specificity | 100 (60.9-100) | 99.4 (96.8-100) | 1.0 (1.0-1.0) | 1.000 |
| Diagnosis according to FNAC: suspicious for malignancy (Bethesda category IV) | | | | |
| - Sensitivity | 82.3 (56.6-96.2) | 83.3 (77.4-88.2) | 1.0 (0.8-1.2) | 1.000 |
| - Specificity | 100 (66.4-100) | 97.6 (91.7-99.4) | 1.0 (1.0-1.1) | 1.000 |
| Diagnosis according to FNAC: non-diagnostic (Bethesda category I) | | | | |
| - Sensitivity | 100 (25.0-100) | 75.0 (30.2-100) | 1.3 (1.0-1.8) | 1.000 |
| - Specificity | 100 (43.8-100) | 98.1 (90.1-99.7) | 1.0 (1.0-1.1) | 1.000 |

^a^ University Medical Center Mainz

^b^ Prospective Evaluation Study Thyroid Surgery 2

^c^ fine-neeedle aspiration cytology
