# Supplementary figures and images for: Targeted use of intraoperative frozen-section analysis lowers the frequency of completion thyroidectomy
Source: BJS Open. 2021 Apr 1;5(2):zraa058. doi: 10.1093/bjsopen/zraa058 (PMC8045471; doi:10.1093/bjsopen/zraa058)

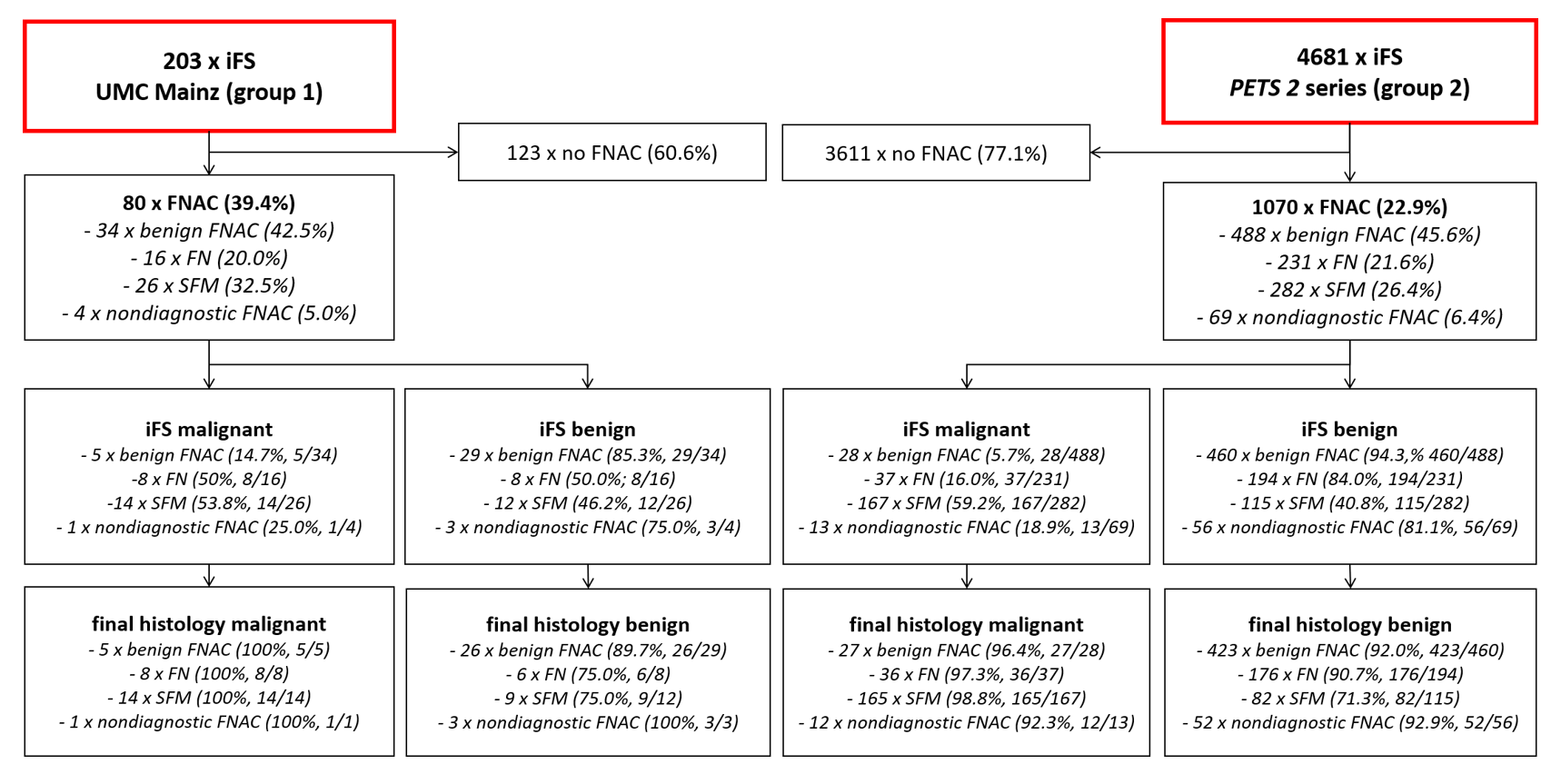

Supplement: zraa058_Supplementary_Data [file zraa058_supplementary_data.zip › Staubitz_Supplementary_Figure_1.tif]
